# Liqui-Tablet: the Innovative Oral Dosage Form Using the Newly Developed Liqui-Mass Technology

**DOI:** 10.1208/s12249-021-01943-w

**Published:** 2021-03-01

**Authors:** Matthew Lam, Kofi Asare-Addo, Ali Nokhodchi

**Affiliations:** 1 Pharmaceutics Research Laboratory, Arundel Building, School of Life Sciences, University of Sussex, Brighton, UK; 2 Department of Pharmacy, University of Huddersfield, Huddersfield, HD1 3DH UK

**Keywords:** Liqui-Tablet, Liqui-Pellet technology, extrusion-spheronization, dissolution enhancement, liquisolid technology

## Abstract

In this study, an attempt was made to produce Liqui-Tablets for the first time. This was carried out through the compaction of naproxen Liqui-Pellets. The incentive to convert the novel Liqui-Pellet into Liqui-Tablet was due to the array of inherent advantages of the popular and preferred tablet dosage form. The study showed that naproxen Liqui-Tablet could be successfully produced and the rapid drug release rate (100% drug release ~ 20 min) could be achieved under pH 1.2, where naproxen is insoluble. It was observed that the different pH of the dissolution medium affected the trend of drug release from formulations with varying amounts of liquid vehicle. The order of the fastest drug-releasing formulations was different depending on the pH used. The presence of Neusilin US2 showed considerable enhancement in the drug release rate as well as improving Liqui-Tablet robustness and hardness. Furthermore, images from X-ray micro-tomography displayed a uniform distribution of components in the Liqui-Tablet. The accelerated stability studies showed acceptable stability in terms of dissolution profile.

## INTRODUCTION

Liqui-Tablet is a newly developed dosage form, stemming from the novel Liqui-Pellet technology, which is also known as the Liqui-Mass technology. Liqui-Tablet is compacted Liqui-Pellet; thus, it is also under the Liqui-Mass system. Note that in previous studies regarding Liqui-Pellet technology, the final dosage form is in a form of Liqui-Pellet filled into a hard-shell capsule (1–5). It is a well-known fact that a tablet is a more commercially favorable dosage form than a capsule in terms of cost-effectiveness. The manufacturing of tablet have lower production costs and higher production rates compared to capsules (6), and costly control steps to ensure capsule integrity are eliminated (7,8). Other advantages of tablets over capsules include the lower tendency of dosage form adhering to the esophagus during ingestion (9); ability to administer higher dose strength than capsule (10); a reduced risk of dosage form being tampered with (6); and an improvement in patient compliance, particularly for those who prefer not to ingest gelatin capsule (11). It is also worth mentioning that the issues with gelatin capsules are not just individual preference but extend to chemical instability (12), the varying dissolution rate of capsule due to varying structure and composition of gelatin (13), and questionable source, particularly from waste leather which may have been treated with harmful substance (14). Hence, there is an incentive to explore the feasibility of Liqui-Tablet.

Since Liqui-Tablet stems from Liqui-Pellet, it is important to understand Liqui-Pellet. In brief, Liqui-Pellet is a combination of concepts from liquisolid technology and pelletization technology. Such a combination can produce a commercially feasible product when using Liqui-Mass system (2), where the active pharmaceutical ingredient (API) is in a liquid state, which is the key feature for the enhanced dissolution rate. One of the key purposes of this technology is to improve the bioavailability of poorly water-soluble drugs by improving its dissolution rate. This is because the bioavailability of poorly water-soluble drugs, particularly biopharmaceutical classification class II (BSC II) drugs, is limited by the API dissolution rate, which affects the absorption rate (15). Liqui-Pellet and Liqui-Tablet’s primary mechanism of improved drug dissolution rate is similar to that of liquisolid formulation, where an increase in surface area available for dissolution, increase in the solubility of API, and improved wettability of drug particles result to enhanced drug release rate (16,17).

**Fig. 1:**
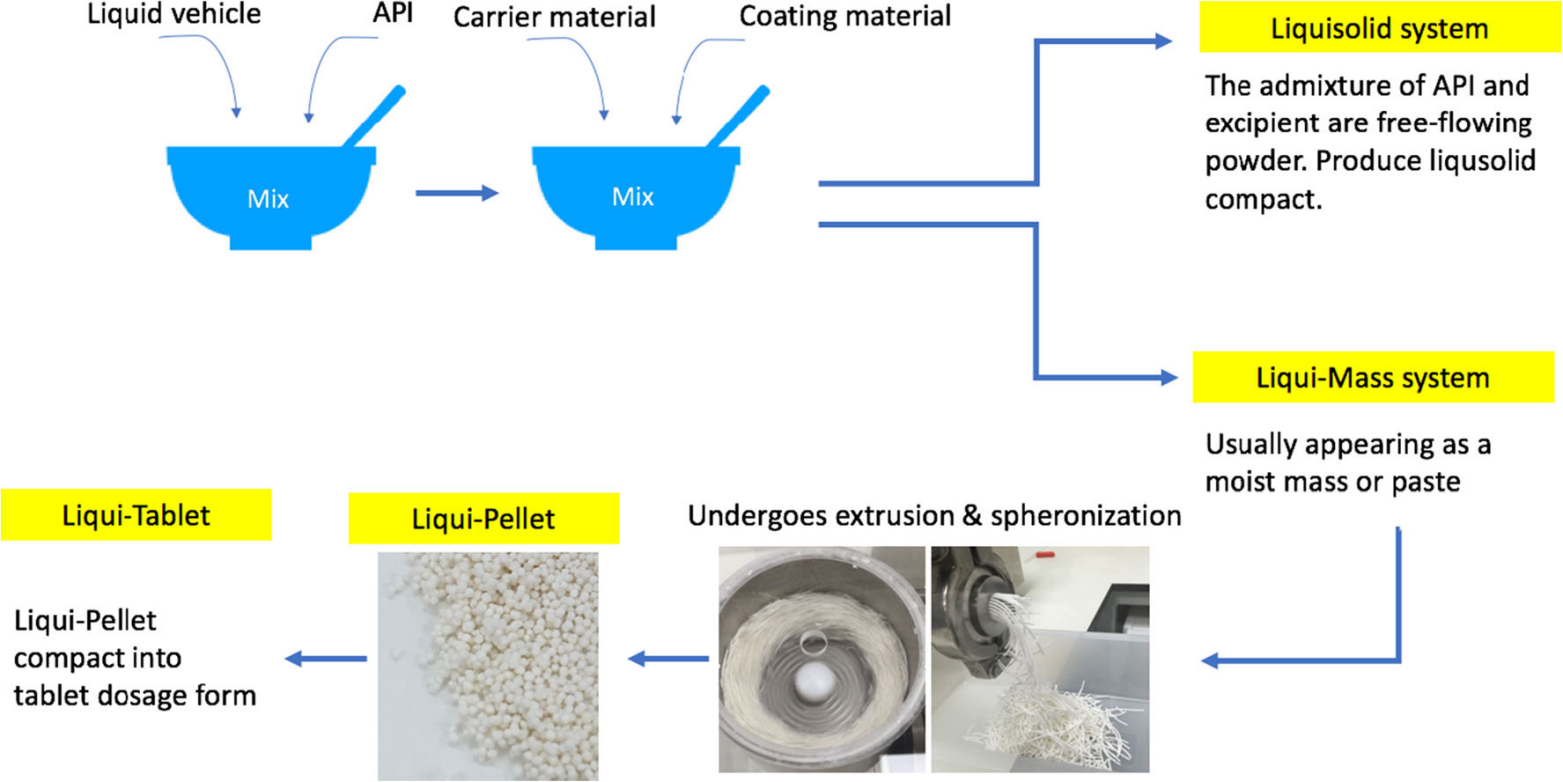
Diagram to differentiate liquisolid system, Liqui-Mass system, Liqui-Pellet, and Liqui-Tablet

Despite Liqui-Pellet technology having similarity to that of liquisolid technology, it is fundamentally different in that it uses Liqui-Mass system as oppose to liquisolid system. This can be seen in Fig. 1 along with the difference between Liqui-Pellet and Liqui-Tablet. However, since Liqui-Pellet technology is a new technology and studies about it is still in its infancy, it may sometimes be mistaken as being the same as liquisolid technology (18). Currently, Lam *et al.* (1,2,4), Lam and Nokhodchi (5), Pezzini *et al.* (19), and Espíndola *et al.* (20) are the only research groups that have started working on the combination of liquisolid and pelletization technology to overcome liquisolid disadvantages. However, the group of Lam *et al.* (1,2,4) and Lam and Nokhodchi (5) uses Liqui-Mass system (belonging to the Liqui-Pellet technology) and the group of Pezzini *et al.* (19) use liquisolid system (belonging to the liquisolid technology). So far such combined technology has been applied to naproxen (1,2,4,5), hydrochlorothiazide (3), ketoprofen (3), ritonavir (20), and felodipine (19).

In order to appreciate the implication of improving the dissolution rate of poorly water-soluble drugs, it is worth pointing out that around 40% of drugs in the market are poorly soluble in the fluid present in the gastrointestinal tract (GIT), which is based on BCS, and around 90% of drugs in development are identified as poorly water-soluble (21). Also, to appreciate Liqui-Mass technology, it should be pointed out that Liqui-Pellet is able to overcome the major drawbacks in liquisolid technology, which is still persisting for more than two decades. These major drawbacks are poor flow properties, poor compressibility, and an inability to produce high-dose drugs of a reasonable size and weight for swallowing (15,16). Liqui-Mass technology overcomes such drawbacks, enabling the concept of liquisolid to be carried forward into a commercially feasible direction.

Since Liqui-Tablet is compacted Liqui-Pellet, it has the same advantages of Liqui-Pellet and arguably more. This is because capsule filling can be removed from the manufacturing process. Liqui-Pellet has key advantages in terms of commercial production and versatile formulation design (3). The potential for commercial production of Liqui-Pellet and Liqui-Tablet is apparent because advanced performing pills can be made using simple approaches where green technology is applicable and advanced preparation and machinery are not required (1,2,4). The production itself is not costly with minimal disruption to current pharmaceutical manufacturing facilities and processes as all equipment and excipients required are commonly found in such facilities (2). In addition, Liqui-Pellet has some key intrinsic advantages from pelletization and liquisolid technologies. It has inherent advantages of pelletization technology/multi-unit pellet form (MUPF) such as good flow property (22); potential to combine incompatible drugs or drugs with different release profiles in same dose unit; flexibility for modification via coating technology; and the reduced likelihood of side effects due to fast gastric emptying and reduced risk of dose dumping as well as being distributed more evenly in the GIT (7,23,24), which also improves bioavailability and reduce variability in drug release (25).

Furthermore, Liqui-Mass technology is capable of versatile formulation design, such as the addition of functional excipients or application of coating technology, while having the API in a liquid state (3). All of these advantageous features make it a unique and interesting approach to the future oral dosage form.

Studies by the authors on naproxen Liqui-Pellet and hydrochlorothiazide Liqui-Pellet (26), which are both poorly water-soluble drugs, have demonstrated remarkably fast drug release rate, ~100% drug release rate in 15 min for both Liqui-Pellet formulations. The dissolution test results from these two Liqui-Pellet formulations are more rapid than solid dispersion, liquisolid compact, and solid self-dispersing mixed micelle forming system.

Despite the advantages of a pellet-based tablet, compaction of pellets into a tablet is a challenging field of research (25,27). The compaction process could lead to pellets fusing into each other, producing a non-disintegrating matrix, which prevents it from reverting into an individual pellet in the GIT; hence, the advantages of multi-unit pellet system (MUPS) would no longer be present (27). Also, the compaction process poses a major challenge for film-coated pellets, where functional coating film is prone to damage and rupture during compression, resulting in unintended changes of the drug release profile (10,25,27,28).

**Fig. 2:**
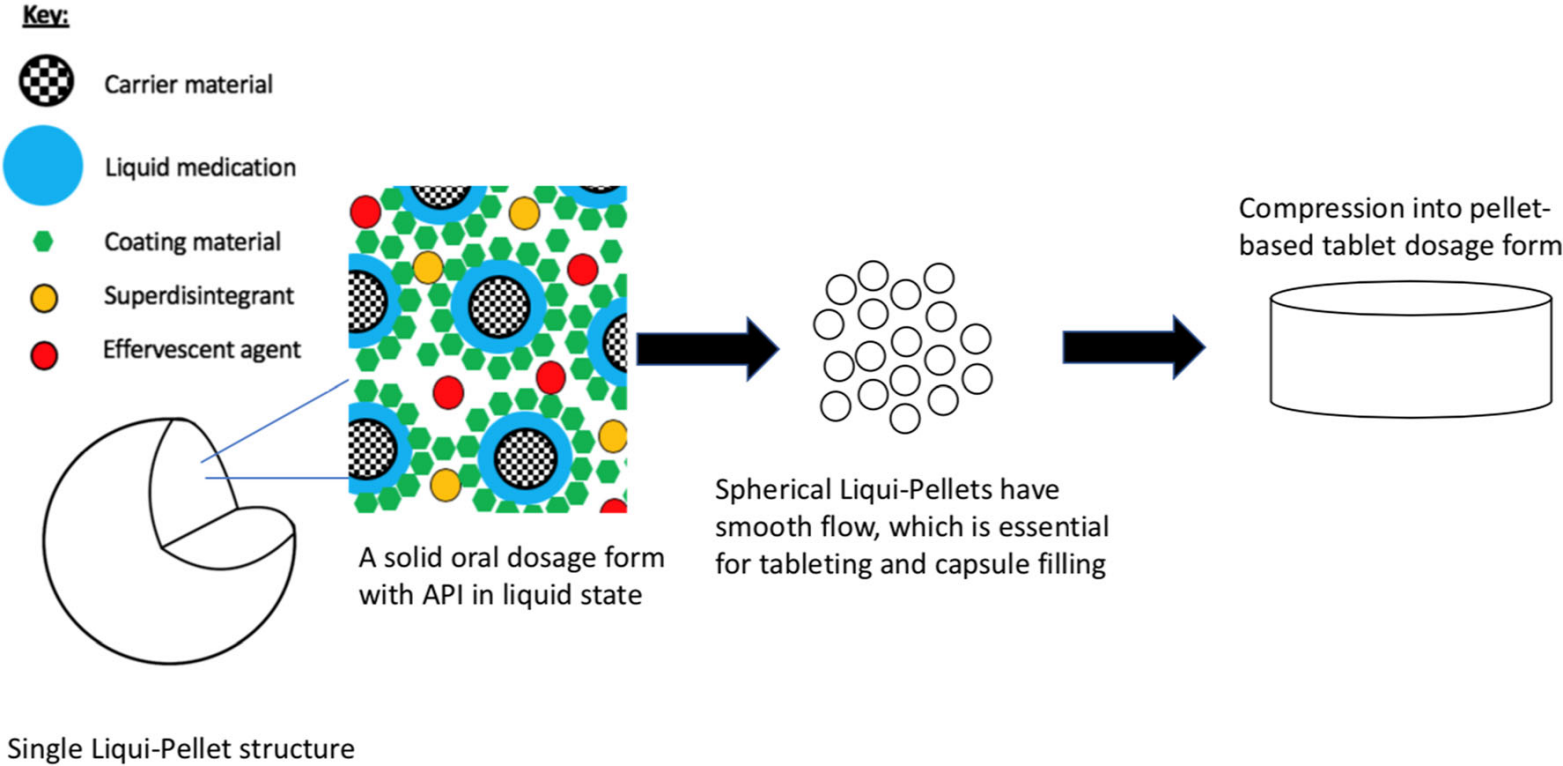
Diagram showing the structure of Liqui-Pellet and how Liqui-Tablet is formed

In this investigation, for the first time, an attempt will be made to study the feasibility of compacting Liqui-Pellets into Liqui-Tablets. The Liqui-Pellets are composed of liquid medication incorporated into a carrier material. The coating material is then added along with additional excipients. These pellets can then be compressed into a pellet-based tablet dosage form, which is shown in Fig. 2. The key objective is to see if Liqui-Tablets can also achieve a rapid drug release like Liqui-Pellet. Fortunately, the Liqui-Pellet that will be studied are not film-coated; therefore, issues concerning the potential rupture of film-coating material will not be present; however, future studies on Liqui-Tablet will explore functional polymeric film coating.

## MATERIALS AND METHODS

### Materials

Materials included naproxen (TCI, Japan), Avicel PH 101 (FMC corp., UK), Aerosil 300 (Evonik Industries AG, Hanau, Germany), Primojel (DFE Pharma, Goch, Germany), Neusilin US2 (Fuji Chemicals, Japan), sodium bicarbonate, (Acros, NJ, USA), and Tween 80 (Acros, Netherlands). All other reagents and solvent were of analytical grades.

### Production of Liqui-Tablets

The Liqui-Tablet formulations were prepared via compacting Liqui-Pellets under a specified compression force using a manual tablet press machine (Compaction model MTCM-I, Globe pharma, UK). All formulations were produced similarly except for the variation in parameters such as carrier composition, Tween 80 concentration, water content, and liquid load factor as shown in Table I. The liquid medication, which is naproxen well mixed in Tween 80, was blended in a specified carrier material alongside with 32% w/w NaHCO_3_ (effervescent agent) and Primojel (superdisintegrant) (Table I). Tween 80 is chosen because it was the most suitable liquid vehicle based on previous studies on Liqui-Pellet (1). The reason why 32% w/w NaHCO_3_ was chosen is that the previous study on effervescent Liqui-Pellet showed that it was the most suitable concentration when considering dosage form total weight and dissolution performance (26). The admixture was mixed for 2 min at a constant rate of 125 rpm using Caleva Multitab (Caleva Process Solutions Ltd, UK). Primojel was added intragranularly because it was observed in previous studies that this led to better disintegration than extragranular incorporation (2). Deionized water of specified amounts was gradually added to achieve a reasonable plastic property for extrusion using Caleva Multitab, which can mix, extrude, and spheronize. This admixture was mixed for a further 5 min before incorporating the Aerosil 300 (coating material). The new admixture was then further mixed for another 5 min before extrusion and spheronization at an almost constant rotation at 4000 rpm (decrease to 2000 rpm if agglomeration seems likely). The duration of spheronization varied depending on the extrudate’s physical property to avoid agglomeration. The moist Liqui-Pellets were then placed in an oven under a constant temperature of 40°C overnight to evaporate excess water content.

**Table I:** Key Formulation Characteristics of the Investigated Liqui-Tablet

Formulation	Water content during extrusion-spheronization (mL) per 20g of admixture of API and excipient	Liquid vehicle concentration (% w/w)	Liquid load factor	Primojel (mg)	Carrier	Mass of carrier (mg)	Mass of coating material (mg)	Compression force (PSI)	Total weight of 25mg naproxen Liqui-Tablet (mg)
					Composition 1^a^, Composition 2^b^				
Physical mixture 1	7.00			5.91	Composition 1	58.06	2.90	400	135.25
Physical mixture 2	7.00			5.91	Composition 1	58.06	2.90	800	135.25
F-1	5.60	19	1	5.92	Composition 1	62.54	3.15	400	197.20
F-2	3.12	23	1.23	7.69	Composition 1	55.06	2.75	400	197.20
F-3	3.20	19	1	5.92	Composition 2	62.54	3.15	400	197.20
F-4	3.20	23	1.23	5.92	Composition 2	55.06	2.75	400	197.20
F-5	3.20	23	1.23	5.92	Composition 2	55.06	2.75	600	197.20
F-6	3.20	23	1.23	5.92	Composition 2	55.06	2.75	800	197.20
F-7	5.60	19	1	5.92	Composition 1	62.54	3.15	800	197.20
F-8	3.12	23	1.23	7.69	Composition 1	55.06	2.75	800	197.20

*Note all formulations contain 25mg of naproxen and 32% w/w NaHCO*_*3*_*, and the carrier to coating material is at a ratio of 20:1*

^*a*^*Carrier composition of 100% Avicel PH101*

^*b*^*Carrier composition of 50% Avicel PH101 & 50% Neusilin US2*

The physical mixture pellet was prepared in a similar manner described above including 32% w/w NaHCO_3,_ but without liquid vehicle incorporated. All formulation’s carrier to coating material ratio was kept constant at 20:1 respectively.

### Pre-Compacted Flowability Test

Flow property of pre-compressed Liqui-Tablet formulations was analyzed using well-established methods, which were also applied to previous studies on Liqui-Mass technology (1,2). The methodology used includes standard flow rate in grams per second, angle of repose, and Carr’s compressibility index (100 taps in 4 min).

### Friability Test on All Formulation

Formulation robustness was investigated using a friabilitor chamber (D-63150, Erweka, Germany). Samples consisting of 10 Liqui-Tablets of specified formulation were set to tumble in the friabilitor machine under constant rotation of 25 rpm for 4 min. The % weight loss of samples was calculated using the weight of samples before and after the tumbling.

### Tablet Hardness Test

All formulations were subjected to tablet hardness tests except for formulations F-2 and F-8, which were too soft for the tablet hardness test. Each formulation was placed in a tablet hardness tester (TBH 125, Erweka, Germany) where the diameter and thickness of each tablet were measured. The tablet hardness tester then measured the amount of force (in Newton) required to fracture the tablet. This was repeated 5 times for each formulation and the mean was calculated.

### Tomography Study

X-ray micro-tomography (XμT) (Nikon XT H 225, Nikon Corp. Tokyo, Japan) was used to assess the tomography of formulation F-5. As formulation F-5 showed the fastest dissolution rate at pH 7.4, it was selected for tomography study. The instrument was set up using a tungsten target, with 90 kV accelerating voltage and an 80 μA gun current. Formulation F-5 was mounted using a double-sided adhesive tape onto a sample stage. Then, a set of 1583 projections was collected from the instrumentation after which these images were reconstructed using CT-Pro, and then examined using a VG Studio 2.1 software.

### *In Vitro Drug Release Test*

All successful formulations were subjected to a dissolution test using the USP paddle method (708-DS Dissolution Apparatus & Cary 60 UV-Vis, Agilent Technologies, USA). The dissolution tests were carried out in such that 900 mL of dissolution medium was at a constant temperature of 37.3 ± 0.5°C. The paddle rotation was set to 50 rpm and the dissolution medium used was either HCl buffer solution at pH 1.2 or phosphate buffer solution at pH 7.4 to simulate fluid in the gastrointestinal tract without enzymes. Spectrophotometric analysis was set to read at wavelength 271 nm at a time interval of 5 min for an hour then a time interval of 10 min for another hour. It should be pointed out that sink condition was not maintained for pH 1.2 and this pH was only used for comparison of various formulations. Under constant temperature of 35°C, naproxen solubility at pH 1.2 was 27 mg/L (29); hence, 25 mg used in test seemed reasonable. However, naproxen is very soluble at pH 7.4 (~3347 mg/L (29); therefore, the sink condition was maintained at this pH.

Dissolution profiles were analyzed using mathematical methods called difference factor (*f*_1_) and similarity factor (*f*_2_). Such mathematical methods have been recommended by the US FDA (Food and drug administration) (30) and implemented by the FDA in various guidance documents (31,32). Difference factor with a value between 0 and 15 and similarity factor with a value between 50 and 100 imply the equivalence of the two dissolution profiles (33).

### Accelerated Stability Test

The accelerated stability test was carried out on formulation F-5, which was one of the fastest drug-releasing Liqui-Tablets in this investigation. The sample was stored at 40°C with a relative humidity of 75%. The duration of the tests was 3 months, where changes in physical appearance and drug release profiles were recorded each month for 3 months.

## RESULTS AND DISCUSSION

### Pre-Compression Flowability Studies on All Formulation

According to flowability results in Table II, there is no issue in terms of flow properties for all formulations. Inference from the angle of repose method shows that all formulations achieve excellent flow properties. There is more variation from the CI; however, there is no issue in flowability as the results ranged from excellent to good flowability. Such results are typical in Liqui-Pellet formulations and further support the claim that this technology has overcome the issue of poor flowability that was prevalent in classical liquisolid technology.

**Table II:** Flow Rate (g/s), Angle of Repose, and Carr’s Compressible Index (CI%) of All Formulations (*n*=3)

Formulation^a^	Flow rate (g/s) ± SD^b^	Angle of repose ± SD^b^	CI% ± SD^b^	Flow property according to angle of repose	Flow property according to CI%
Physical mixture 1	8.75 ± 0.19	24.39 ± 0.56	13.32 ± 0.00	Excellent	Good
Physical mixture 2	8.75 ± 0.19	24.39 ± 0.56	13.32 ± 0.00	Excellent	Good
F-1	8.10 ± 0.17	26.71 ± 0.20	10.23 ± 0.00	Excellent	Excellent to good
F-2	7.81 ± 0.28	28.92 ± 0.49	10.33 ± 1.14	Excellent	Excellent to good
F-3	7.86 ± 0.19	28.58 ± 1.00	11.17 ± 0.00	Excellent	Good
F-4	8.37 ± 0.11	26.83 ± 0.79	10.23 ± 0.00	Excellent	Excellent to good
F-5	8.37 ± 0.11	26.83 ± 0.79	10.23 ± 0.00	Excellent	Excellent to good
F-6	8.37 ± 0.11	26.83 ± 0.79	10.23 ± 0.00	Excellent	Excellent to good
F-7	8.10 ± 0.17	26.71 ± 0.20	10.23 ± 0.00	Excellent	Excellent to good
F-8	7.81 ± 0.28	28.92 ± 0.49	10.33 ± 1.14	Excellent	Excellent to good

^*a*^*For the composition of each formulation, refer to Table* *I*

^*b*^*SD, standard deviation from the mean*

### Studies on Liqui-Tablet Robustness

The compaction of Liqui-Pellets into Liqui-Tablet was successful; however, not all formulations passed the friability test, which suggests that some formulations are not robust enough (Table III). Physical mixture 1 and formulations F-1, F-2, F-7, and F-8 all fractured, thus, failing the friability test. Interestingly, all of those failed formulations did not contain Neusilin US2, and all of the formulations that passed the friability test contained Neusilin US2. Hence, it seems that the carrier composition is an important factor to consider in Liqui-Tablet production. It is speculated that the extremely large specific surface area of Neusilin US2 (an amorphous form of magnesium aluminometasilicate), which is 300 m^2^/g (34), may have contributed to the sufficient bonding strength upon compaction of the Liqui-Pellets; hence, Liqui-Tablets containing Neusilin US2 were robust enough to pass the friability test. In addition, tablet hardness test results (Table IV) showed that formulations containing Neusilin US2 have increased hardness.

**Table III:** Friability Test Results of All Formulations

Formulation^a^	% weight loss	Fractured (yes/no)	Pass/fail
Physical mixture 1	NA^b^	Yes	Fail
Physical mixture 2	0.15	No	Pass
F-1	NA^b^	Yes	Fail
F-2	NA^b^	Yes	Fail
F-3	0.00	No	Pass
F-4	0.00	No	Pass
F-5	0.00	No	Pass
F-6	0.00	No	Pass
F-7	NA^b^	Yes	Fail
F-8	NA^b^	Yes	Fail

^*a*^*For the composition of each formulation, refer to Table* *I*

^*b*^*Not applicable*

**Table IV:** Tablet Hardness Test Results of All Formulations

Formulation^a^	Mean thickness ± SD^b^ (mm)	Mean diameter ± SD^b^ (mm)	Mean hardness ± SD^b^ (N)
Physical mixture 1	5.98 ± 0.05	5.23 ± 0.02	56.80 ± 10.94
Physical mixture 2	5.60 ± 0.01	5.25 ± 0.01	102.60 ± 13.03
F-1	7.92 ± 0.05	5.25 ± 0.01	85.20 ± 8.11
F-3	7.55 ± 0.02	5.26 ± 0.01	90.40 ± 2.70
F-4	7.66 ± 0.02	5.26 ± 0.00	54.60 ± 3.13
F-5	7.61 ± 0.03	5.25 ± 0.00	60.60 ± 5.27
F-6	7.60 ± 0.02	5.27 ± 0.01	52.40 ± 2.51
F-7	7.87 ± 0.02	5.26 ± 0.01	73.60 ± 5.59

^*a*^*For the composition of each formulation, refer to Table* *I*

^*b*^*SD, standard deviation from the mean*

Both physical mixtures 1 and 2 have the same composition; the only difference is that different compression forces were applied, 400 PSI and 800 PSI respectively. Physical mixture tablet 1, which was made using lower compression force than physical mixture tablet 2, failed the friability test, and physical mixture tablet 2 passed. This indicates that compaction force influences the physical property of the physical mixture tablet and that higher compaction force results in a more robust tablet.

All Liqui-Tablets which contain Neusilin US2 passed the friability test despite the differences in liquid vehicle concentration and compression force. Thus, Neusilin US2 seems to be the single most important factor in Liqui-Tablet that influences the dosage form robustness. These Liqui-Tablet formulations have 0% weight loss after being subjected to the friabilitor, which is due to the plastic property of the formulations. This plastic property makes the tablet resistant to friability.

Although some formulations failed the friability test due to fracturing of the Liqui-Tablet, it should be noted that there are simple approaches to overcome this issue such as incorporating binding excipient, manipulating the pre-compressed Liqui-Tablet physical properties, or incorporating a mixture of excipient-based pellet with the Liqui-Pellet. Such modifications will be carried forward in future investigation.

### Tablet Hardness Test

The tablet hardness test results (Table IV) show that the compression force has major influences on the hardness of the physical mixture tablet, but interestingly have hardly any influences on Liqui-Tablet formulations. Physical mixtures 1 and 2 have the same composition, but physical mixture 2 was compressed with double the amount of force compared to physical mixture 1 (800 PSI and 400 PSI respectively), which results to physical mixture 2 having around twice the hardness of the physical mixture 1 compacts (56.8 N and 102.6 N respectively). However, in the case of Liqui-Tablets, the compression force seems to have hardly any effect on its hardness. This can be seen in formulations F-1 and F-7, where both formulations have the same composition (Table I), but F-7 was compressed with twice as much force than F-1 (800 PSI and 400 PSI respectively). Despite the difference in compression force, both F-1 and F-7 hardness are not very much different (85.2 N and 73.6 N respectively). A similar observation was made for formulations F-4, F-5, and F-6 where their composition is the same but the compression force differs (400 PSI, 600 PSI, and 800 PSI respectively), but their tablet hardness are similar (54.6 N, 60.6 N, and 52.4 N respectively).

It is observed that the amount of liquid vehicle in the Liqui-Tablet formulation has major influences on Liqui-Tablet hardness. By increasing liquid vehicle concentration, the hardness of Liqui-Tablet is reduced. This is shown in formulations F-3 and F-4 where both compositions are the same except for the amount of liquid vehicle (concentration of Tween 80 of 19% w/w and 23% w/w respectively). With the higher concentration of Tween 80 in F-4 in comparison to F-3, the tablet hardness is reduced considerably (54.6 N and 90.4 N respectively). The influences of the liquid vehicle are also shown in F-1 and F-2, where both formulations are identical except that F-2 has a higher concentration of Tween 80 than F-1, which results to F-2 being too soft for the tablet hardness test. A similar observation is made for F-7 and F-8, where F-8 having a higher amount of Tween 80 is too soft for tablet hardness test to be carried out. Hence, liquid vehicle concentration is one of the major parameters that determine Liqui-Tablet hardness.

Another observed parameter that has major influences on the Liqui-Tablet hardness is the carrier composition. Formulations containing Neusilin US2 increase Liqui-Tablet hardness. This is shown in F2 and F4 where both have the same high concentration of Tween 80 (23% w/w) and compressed at the same force (400 PSI); however, only F-2, which is absent of Neusilin US2, is too soft for hardness test to be applied. The same observation is made in F-8 and F-6, where F-8, which does not contain Neusilin US2, is too soft to be tested by the tablet hardness tester.

Overall, liquid vehicle concentration and carrier composition are important factors to consider in terms of Liqui-Tablet hardness. As for the compression force, it does not seem to have any observable effect on Liqui-Tablet hardness.

### Tomography

**Fig. 3:**
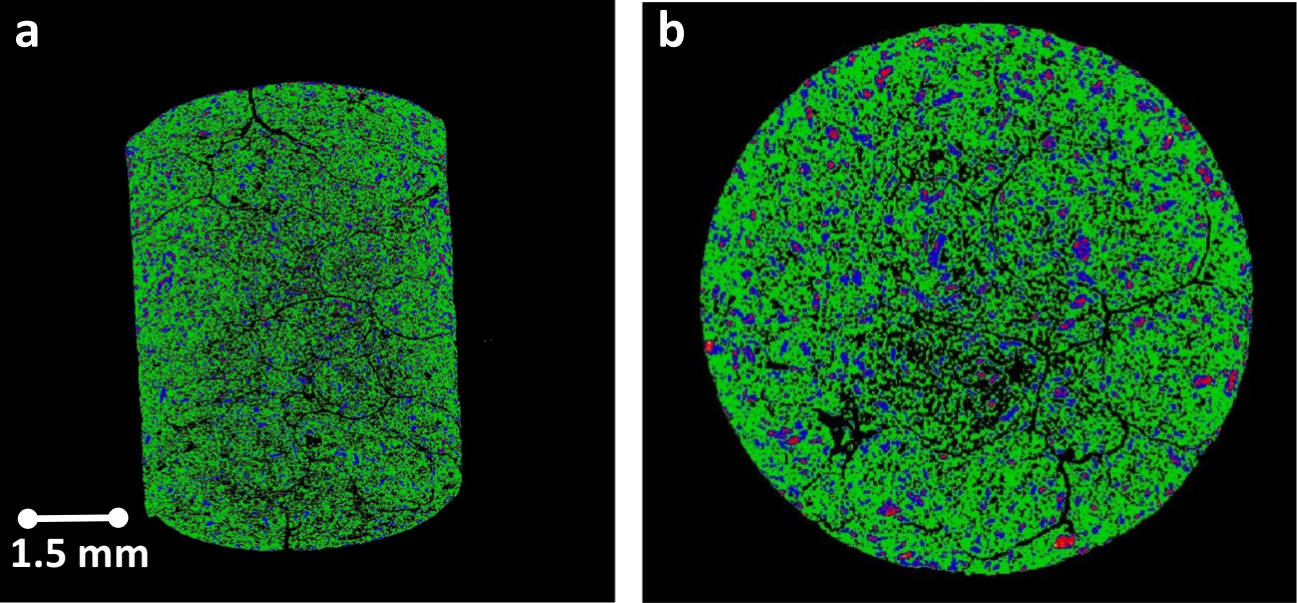
X-ray micro-tomography of the sagittal and diametric images of compacted F-5 Liqui-Tablet formulation **a** side of the tablet **b** top of the tablet. Note: This tomography technique is based on the differential absorbance of X-rays between materials of differing electron density. The color code is therefore from the density histogram. The green coloration represents the bulk of the material from the formulation which for F5 is the carrier composition of 50% Avicel PH101 & 50% Neusilin US2. The pink coloration represents the naproxen content with the blue color surrounding it representing the liquid vehicle. The black coloration within the Liqui-Tablet representing the pore spaces as well the demarcations of the individual pellets compressed together to form the Liqui-Tablet

X-ray tomography of F-5 is shown in Fig. 3. The black spaces are the porosity within the compact. The continuous black lines show the individual pellets that are compressed to make the compact. The different colorations in these figures are assigned to the different components that made the F-5. By looking at the formulation table and the amount/content of each excipient used, it is possible to determine which color belongs to which excipient. Generally, it can be said the figure shows the components in the Liqui-Tablet are distributed uniformly.

### *In Vitro Dissolution Test*

**Fig. 4:**
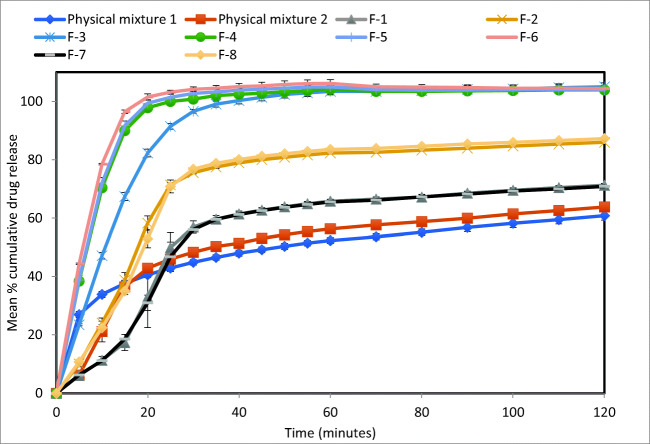
Dissolution profiles of all formulations at pH 1.2

The dissolution test results of all formulations under acidic conditions (pH 1.2), which is used to mimic the stomach condition, are shown in Fig. 4. The results show a trend that increasing liquid vehicle concentration results to increase in drug release rate. This is shown when comparing similar formulations with different liquid vehicle concentration such as F-1 (Tween 80 19% w/w) and F-2 (Tween 80 23% w/w), where F-2 have ~17% more drug release after 2 h than F-1 (*f*_1_= 23.61 and *f*_2_= 38.89). This is also observed in other formulations such as F-7 (Tween 80 19% w/w) and F-8 (Tween 80 23% w/w), where F-8 have ~17% more drug release after 2 h than F-7 (*f*_1_= 24.55 and *f*_2_= 38.03); and F-3 (Tween 80 19% w/w) and F-4 (Tween 80 23% w/w), where F-4 have ~4% more drug release after 2 h than F-3 (*f*_1_= 8.71 and *f*_2_= 47.52). Such observation is in agreement with the authors’ previous studies on the crucial effect of co-solvent in Liqui-Pellet (4), which also contains solid-state analysis on the naproxen Liqui-Pellet. In general, XRPD and DSC data showed naproxen Liqui-Pellets have a reduced crystallinity in comparison to the physical mixture pellet.

It is expected that the increase in non-volatile co-solvent increases the proportion of API in the molecular state in the liquid vehicle, which increases the diffusion of API from Liqui-Pellets to the dissolution medium (17). Previous saturation solubility study (under constant agitation at 37°C for 72 h) showed that naproxen solubility in Tween 80 was 21.85 mg/mL, which is considered sparingly soluble (4). Therefore, it would make sense that more Tween 80 would result in more naproxen being solubilized. Naproxen is only partially dissolved in Tween 80, which was determined using the mentioned saturated solubility data and Tween 80 density (1.06 g/cm3). It is estimated that the formulation containing 19% Tween 80 has 25 mg of naproxen in ~0.035 mL Tween 80, and 25 mg of naproxen in ~0.043 mL for the formulation with 23% Tween 80. Furthermore, since Tween 80 reduces surface tension/cohesive force, the higher the amount of Tween 80 the greater the extent of disintegration, thus, further enhancing the drug release. A similar finding in terms of Tween 80 improving propensity of the disintegration of MCC-based pellet was observed by Chamsai and Sriamornsak (35).

When comparing formulations with identical composition but under different compression forces, there is no observable effect on the drug release rate. This can be seen when comparing F-1 and F-2/F-7 and F-8/F-4/F-5 and F-6. Unlike a compressed powdered tablet, where the compression force can significantly influence the propensity of disintegration and consequently drug release rate, the Liqui-Tablet reverts back to pellet form under a minute; hence, there is less variation in disintegration and drug dissolution rate for Liqui-Tablet made under different compression force.

Formulations with Neusilin US2 have significantly faster drug release rate than formulations absent of Neusilin US2. This is shown when comparing formulation F-1 and F-3, where both have the same liquid vehicle concentration and compression force, but F-3 has a significantly faster drug release rate due to the presence of Neusilin US2. Formulation F-3 reached 100% drug release after ~45 min, whereas F-1 only reached ~71% drug release after 2 h (*f*_1_= 41.17 and *f*_2_= 21.51). A similar observation is made in formulations F-2 and F-4, where F-4 drug release rate is considerably faster due to the presence of Neusilin US2. Formulation F-4 start plateauing at 100% drug release at around 25 min, whereas F-2 only reached 86% drug release after 2 h (*f*_1_= 27.18 and *f*_2_= 28.33). It is speculated that the increase in dissolution rate could be due to a possible naproxen complexation with Neusilin. In a study by Gupta *et al.* (36), it was reported that amorphization of naproxen (including other acidic API) was formed during ball milling with Neusilin, which was supported through X-ray powder diffraction and differential scanning calorimetry. It was mentioned that the drug hydrogen bonds onto the extensive surface of Neusilin, reducing the crystalline phase of the drug. The complexation of the amorphous state of naproxen was due to an acid-base reaction between carboxyl moiety of naproxen and the Neusilin silanol or due to ion-dipole interactions between metal ions in Neusilin and the drug. Due to the higher energy of the amorphous phase, the drug solubility and dissolution rate increased. In the case of Liqui-Tablet, the naproxen was not milled but the stress induced by liquid vehicle and extrusion process likely reduces the crystalline phase. The naproxen that can potentially recrystallize out of the liquid medication or those not fully solubilized in Tween 80 may complex with Neusilin US2 to form a stable amorphous state, therefore enhancing dissolution rate. However, more investigation is required to confirm this.

On comparing the dissolution profile of formulation F-6, which displayed one of the fastest drug release rate in this study, with the fastest drug-releasing naproxen Liqui-Pellet from the author’s previous work (37), there is no observable difference in the drug release profile at pH 1.2, both plateau to 100% after 20 min. The effervescent agent in the Liqui-Tablet interacts with the acidic dissolution medium, which produces CO_2_ gas, promoting disintegration and a very fast transition from tablet to pellet form. However, in an alkaline environment (pH 7.4), the Liqui-Tablet drug release is slower than the Liqui-Pellet in a capsule. This is because the effervescent agent is not triggered at this alkaline pH.

**Fig. 5:**
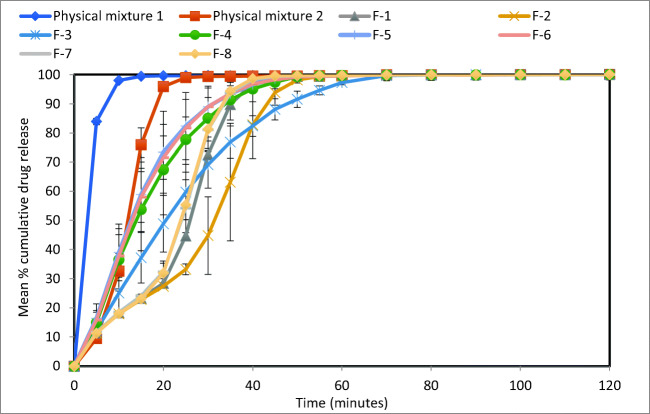
Dissolution profiles of all formulations at pH 7.4

The dissolution test results in alkaline condition (pH 7.4) mimic the small intestine pH condition where naproxen is soluble (Fig. 5). It is surprising to see that the physical mixture Tablets I and II had a faster drug release rate than the Liqui-Tablet formulations. It is postulated that since the API is soluble at this alkaline pH, the rate-limiting step for drug release is the disintegration rate and surface area available for drug release. If that is the case, then perhaps the liquid vehicle in Liqui-Tablet is reducing the propensity of disintegration as it could be acting as a binding material, hence, displaying slower drug release. Such postulation comes from data in Table IV, where it can be seen that under compression force 400 PSI, the formulation with Tween 80 (F-1 = 85.2 N and F-2 = 90.4 N) is harder than the physical mixture (56.8N). This postulated binding action on drug release is not observed at pH 1.2 because the effervescent agent is triggered in an acidic environment. The results from Fig. 5 also show that compression force does not have a major effect on Liqui-Tablet drug release rate as seen in formulations F-4, F-5, and F-6, where all of these formulations’ composition are the same but the compression force applied to them are different (400 PSI, 600 PSI, and 800 PSI respectively).

Overall, varying compaction force does not seem to have any influences on Liqui-Tablet drug release rate. Naproxen Liqui-Tablet drug release at acidic pH is considered very fast (F-6 starting to plateau at 20 min) and more rapid than naproxen solid dispersion (38) and naproxen liquisolid compact (39).

The concentration of liquid vehicle and the presence of Neusilin US2 have major influences on the drug release rate at acidic pH. As expected, the increase in liquid vehicle concentration improves the enhancement of drug release at pH 1.2. It is noteworthy to point out that the formulation with the highest amount of Tween 80 (23% w/w or 45.35mg per dose unit) is below Tween 80’s maximum potency per unit dose for oral tablet according to US FDA, which somewhat reflects the safety potential for commercial manufacturing of such dosage forms.

It is interesting to observe that at pH 7.4, the liquid vehicle in Liqui-Tablet could be responsible for slowing down the drug release rate possibly due to binding action, which reduces the propensity for disintegration. The presence of Neusilin US2, which is part of the carrier material, improves the drug release rate considerably. Not only does Neusilin US2 improves drug release rate but it also improves the robustness and hardness of Liqui-Tablet (Tables III and IV), making it a valuable excipient in this technology. Future studies will include investigating different excipients that may improve Liqui-Tablet quality and drug release performance.

### Accelerated Stability Studies

**Fig. 6:**
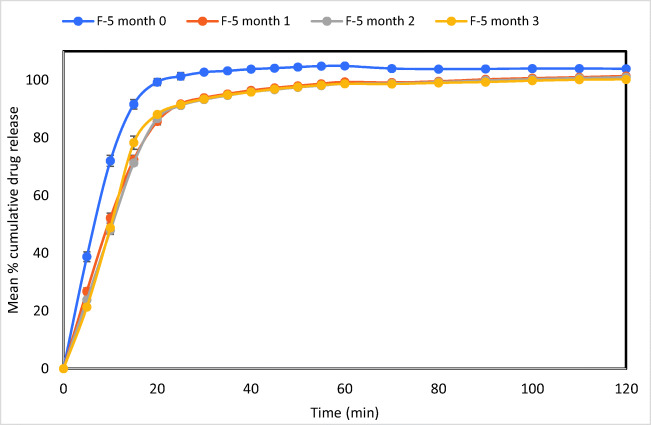
Stability test of formulation F-5 represented through dissolution profile taken each month over the period of 3 months under pH 1.2

The drug release rate of formulation F-5 (Fig. 6) was investigated under stress conditions for the accelerated stability test over 3 months. In comparing F-5 drug dissolution profile at month 0 and a month afterwards (month 1), there is a difference in the dissolution profile (*f*_1_ = 22.52 and *f*_2_ = 42.61), indicating some degree of decrease in drug release. This difference in dissolution profile may be due to changes in the formulation over storage time such as pellet becoming harder over time. The degradation becomes less apparent after the first month where F-5 drug dissolution profile in month 1 and month 2 shows *f*_1_ = 3.03 and *f*_2_ = 80.61. A similar observation is made between month 2 and month 3 where *f*_1_ = 5.01 and *f*_2_ = 72.46.

## Conclusion

The Liqui-Tablet formulation was successfully manufactured for the first time using naproxen as the drug model. In comparison to the rapid drug release of naproxen Liqui-Pellet from previous studies, it can be concluded that the rapid drug release is maintained in Liqui-Tablet dosage form at acidic pH. Nonetheless, when comparing the dissolution profile of the best naproxen Liqui-Tablet with other studies concerning naproxen solid dispersion and liquisolid compact, the drug release rate of Liqui-Tablet is more rapid. It is interesting how the liquid vehicle in naproxen Liqui-Tablet under pH 7.4 actually slows down the drug release rate. This is possibly due to liquid vehicle binding effect within the Liqui-Tablet, which reduces the propensity for disintegration. The disintegration step seems to be the rate-determining step at this pH as API solubility is no longer an issue. Compression force show little influence on Liqui-Tablet hardness; however, liquid vehicle concentration has a considerable impact on Liqui-Tablet hardness.

The studies also confirm that compaction force during tableting has no observable effect on the Liqui-Tablet drug release rate. The presence of Neusilin US2 in Liqui-Tablet formulations has shown to be an important factor to achieve ideal Liqui-Tablet physical properties such as robustness and hardness, as well as allowing faster drug release rate to be achieved. The improved drug release rate may be due to amorphous naproxen and Neusilin US2 complex, but require further investigation to confirm this. Furthermore, accelerated stability studies have shown acceptable stability.
